# On the role of the sorting platform in hierarchical type III secretion regulation in enteropathogenic *Escherichia coli*

**DOI:** 10.1128/jb.00446-24

**Published:** 2025-03-03

**Authors:** Arely Marcos-Vilchis, Norma Espinosa, Adrián F. Alvarez, José L. Puente, J. Eduardo Soto, Bertha González-Pedrajo

**Affiliations:** 1 Departamento de Genética Molecular, Instituto de Fisiología Celular, Universidad Nacional Autónoma de México 61739 , Mexico City, Mexico; 2 Departamento de Microbiología Molecular, Instituto de Biotecnología, Universidad Nacional Autónoma de México, Morelos, Mexico; 3 Programa de Ingeniería Genómica, Centro de Ciencias Genómicas, Universidad Nacional Autónoma de México, Morelos, Mexico; University of Virginia School of Medicine, Charlottesville, Virginia, USA

**Keywords:** EPEC, T3SS, sorting platform, hierarchical secretion, injectisome, gatekeeper complex

## Abstract

The virulence of enteropathogenic *Escherichia coli* (EPEC) depends on a type III secretion system (T3SS), a membrane-spanning apparatus that injects effector proteins into the cytoplasm of target enterocytes. The T3SS, or injectisome, is a self-assembled nanomachine whose biogenesis and function rely on the ordered secretion of three distinct categories of proteins: early, middle, and late type III substrates. In EPEC, this hierarchical secretion is assisted by several cytosolic protein complexes at the base of the injectisome. Among these, the sorting platform is involved in the recognition and sequential loading of the different classes of T3-substrates. In addition, a heterotrimeric gatekeeper complex, also known as a molecular switch, operates in concert with components of the T3SS export apparatus to guarantee the delivery of middle substrates prior to late substrate secretion. In this study, we showed that the sorting platform is differentially required for the secretion of distinct categories of substrates. Moreover, we demonstrated a cooperative interplay and protein–protein interactions between the sorting platform and the gatekeeper complex for proper middle and late substrate docking and secretion. Overall, our results provide new insights into the intricate molecular mechanisms that regulate protein secretion hierarchy during T3SS assembly.

**IMPORTANCE:**

Enteropathogenic *Escherichia coli* employs a type III secretion system to deliver virulence proteins directly into host cells, disrupting multiple cellular processes to promote infection. This multiprotein system assembles in a precise stepwise manner, with specific proteins being recruited and secreted at distinct stages. The sorting platform and the gatekeeper complex play critical roles in regulating this process, but their cooperative mechanism has not been fully elucidated. Here, we reveal a novel functional interaction between these two components, which is critical for hierarchical substrate recognition and secretion. These findings advance our understanding of the molecular mechanisms underlying bacterial virulence and suggest new potential targets for antimicrobial strategies aimed at disrupting T3SS function.

## INTRODUCTION

Bacterial colonization is a remarkable case of adaptation, enabling microorganisms to replicate in privileged ecological niches within eukaryotic hosts. To accomplish this, bacteria have acquired a suite of sophisticated strategies, including the type III secretion system (T3SS), a nanomolecular machine known as the injectisome, which allows the direct delivery of effector proteins into the cytoplasm of host cells (1, 2). The T3SS is used by many human pathogenic bacteria to inject multiple effectors that subvert a wide variety of host cell processes and signaling pathways. This coordinated assault suppresses host cell defenses and promotes bacterial establishment (1, 3).

Enteropathogenic *Escherichia coli* (EPEC) is an etiological agent of infant diarrhea and one of the leading causes of mortality in children under 5 years of age in the Global South (4, 5). EPEC, along with enterohemorrhagic *E. coli* (EHEC), *E. albertii,* and *Citrobacter rodentium*, belongs to the attaching and effacing (A/E) family of pathogens that encode a T3SS on a pathogenicity island known as the locus of enterocyte effacement (LEE) (6). The effectors translocated by the EPEC T3SS promote tight bacterial attachment to the intestinal epithelium, effacement of adjacent microvilli, and localized actin accumulation beneath the bacterial–host interface, forming a histological injury called A/E lesion, which facilitates colonization of the gut mucosa (7, 8).

The T3SS structure consists of nearly 20 distinct proteins, many of which display a high degree of conservation across different bacterial species and with the bacterial flagellum (9, 10). Throughout this manuscript, we will use the Sct (*secretion and cellular translocation*) unified nomenclature for injectisome-type T3SSs (11) and the EPEC-specific nomenclature when referring to distinctive proteins in this bacterium.

The hallmark that defines the T3SS is its syringe-shaped structure, organized into three key structural modules (Fig. S1): (i) a cytoplasmic sorting platform consisting of the SctK, SctQ, and SctL proteins (hereafter SctKQL), for protein recruitment and sequential loading, and an ATPase complex (SctNO), for chaperone dissociation and substrate unfolding (12–14); (ii) a basal body consisting of different ring assemblies, two concentric rings embedded in the inner bacterial membrane (SctDJ) and a secretin ring (SctC) in the outer membrane, which anchor the entire secretion machinery to the cell envelope; and (iii) a hollow needle-like structure protruding beyond the bacterial surface, formed by the helical polymerization of the SctF protein (10). The needle is capped with a tip protein (SctA), which assists insertion of the translocation pore (SctBE) into the host cell membrane, providing a continuous conduit for the transit of effectors (15–17). In A/E pathogens, the needle tip protein extends into a long filamentous structure with an internal lumen of 2.2 nm, formed by the helical assembly of subunits of the EspA protein (18). Situated within the basal body, the inner membrane rings house the export apparatus components (SctRSTUV), which serve as the initial entry point into the secretion channel. The needle adapter (or inner rod), formed by subunits of the SctI protein, connects the export apparatus core proteins (SctRST) with the needle (19, 20). The SctUV membrane proteins have prominent cytosolic domains that are involved in T3 substrate recognition, thus acting as an export gate (21).

The biogenesis of the T3SS follows a hierarchical process, relying on the sequential secretion of different categories of T3 substrates (22). Early substrates (SctI and SctF) are secreted and assembled first, enabling the subsequent secretion of middle substrates or translocators (SctABE) through the SctI needle adapter and the SctF needle to assemble the translocon. Once the translocation pore is embedded into the host cell membrane, a shift in secretion substrate specificity allows the passage of late substrates or effectors directly into the host cell (8, 23–25). The first substrate specificity switch occurs when the molecular ruler protein (SctP) detects that the needle has reached its final length and interacts with the SctU export gate component, stopping secretion of early substrates (26–29). The progression from middle to late substrate secretion is controlled by a second molecular switch, known as the gatekeeper complex (or SctW complex), which in EPEC is made up of the SepL(SctW)/SepD/CesL proteins (30–32). This complex associates with the export gate protein SctV, favoring its ability to recognize middle substrates with high affinity, while impeding the recognition of late substrates (31–33). Once the translocation pore is inserted into the target cell, this event signals the export apparatus through a mechanism that is not yet fully understood (34–38), releasing the repression exerted by the SctW complex and allowing SctV to recognize late substrates (32).

The secretion hierarchy is further facilitated by a large cytosolic complex known as the sorting platform (SP). This complex acts as a physical scaffold where different categories of T3 substrates are loaded and sorted prior to secretion (13). Biochemical studies conducted in *Salmonella enterica* have shown that in injectisomes primed for protein injection, the SP is predominantly occupied by middle substrates. Conversely, in a genetic background lacking translocators, the SP shifts to engage late substrates, suggesting that an ordered protein secretion may depend on different binding affinities of substrates or chaperone–substrate complexes to SP components (13). Cryo-electron tomography studies in *Shigella* (39) and *Salmonella* (40) have shown that the SP forms a cage-like structure composed of six pods radially arranged around the cytoplasmic portion of the export gate (Fig. S1). This assembly is formed by the SctK protein, which connects the entire cytosolic complex to the basal body and interacts with SctQ, the main constituent of the pods, which in turn serves as a scaffold for the cradle-like structure made up by the protein SctL. Within this structural framework, SctL accommodates the SctN and SctO ATPase complex in close proximity to the entry of the SctV export gate (39–43).

The absence of any SP component prevents assembly of the needle structure and translocation pore, thereby leading to a nonfunctional T3SS (39–41, 44, 45; Fig. S2). However, we have shown that increased levels of middle and late substrates can bypass the requirement of an intact SP, suggesting that this structure may also function as a docking platform to recruit these substrates and increase their local concentration near the export gate (12, 44, 45). Additionally, the SP is dynamic since its components have been shown to alternate between being associated with the injectisome and existing as soluble proteins and subcomplexes in the cytosol, with subunit exchange between both states (46). In a more recent study, SctQ and SctL were shown to bind effectors in live bacteria, and a shuttling model was proposed in which these proteins, or their different cytosolic complexes, deliver effectors to the injectisome structure (47).

While two major protein complexes, the SP and the gatekeeper complex, are directly involved in the hierarchical secretion of substrates, it remains unclear how they coordinate to ensure timely substrate delivery. Moreover, it is uncertain whether these complexes also regulate the secretion of early substrates. In this study, we exploited a substrate overproduction assay in SP and gatekeeper complex mutants to demonstrate that, in contrast to middle and late substrates, the SP is strictly essential for early substrate secretion. Additionally, we found that these two subcomplexes interact to form a larger modular complex and functionally cooperate in substrate recognition. Based on these findings, we propose a model for hierarchical substrate recruitment, providing new insights into the early stages of T3 protein engagement.

## RESULTS

### Sorting platform bypassing distinguishes secretion hierarchy

**Fig 1:**
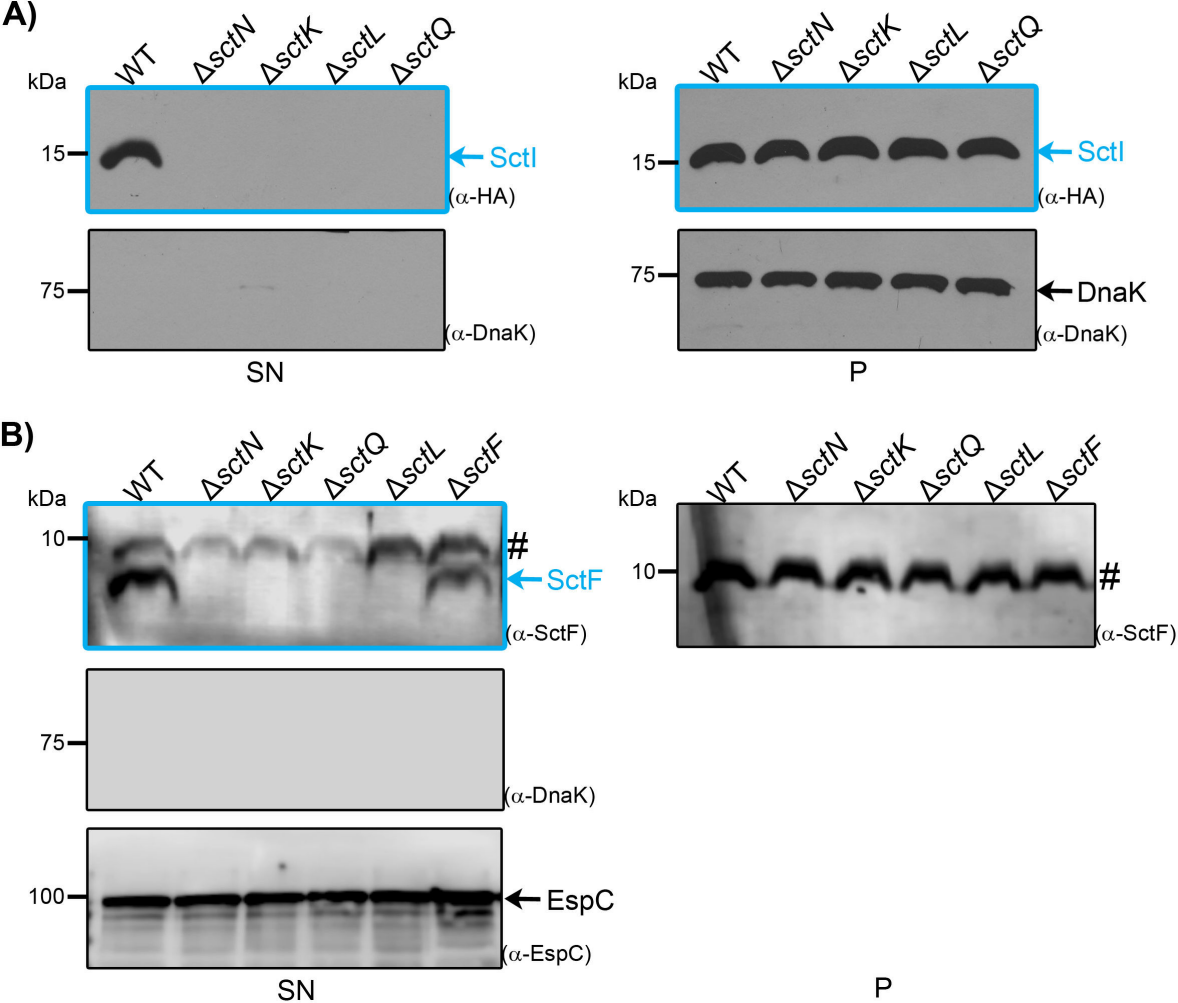
Overproduction of early substrates cannot overcome the lack of a sorting platform. Wild-type EPEC (WT) or the indicated nonpolar mutant strains carrying either (**A**) a high-copy plasmid (pTOPO2HA) expressing C-terminally HA-tagged SctI or (**B**) a middle-copy plasmid (pTrc99AFF4) expressing untagged SctF were grown under T3SS-inducing conditions. The presence in the supernatant (SN) or in the whole-cell lysate (**P**) of the early substrates (**A**) SctI or (**B**) SctF was monitored by immunodetection with anti-HA or polyclonal anti-SctF antibodies, respectively. (**A**) The cytosolic chaperone DnaK served as a loading control for bacterial whole-cell lysates and as a cell lysis control in the bacterial supernatant. The autotransporter protein EspC served as a supernatant loading control. Results are representative of three independent biological replicates. Molecular mass markers are shown on the left (kDa). The hashtag on the anti-SctF blots denotes an unspecific band. Early substrates are color-coded in blue.

We previously developed an *in vitro* secretion assay in which individual substrates were overproduced in sorting platform-deficient EPEC strains, revealing that the overexpression of various middle and late T3 substrates is sufficient to bypass the lack of a complete SP (12). However, it remains unknown whether this effect extends to early substrates. To further investigate the role of the SP in orchestrating type III secretion, we extended our analysis to the two early substrates SctI and SctF. The coding regions of *sctI* and *sctF* were cloned into the high-copy-number vector pTOPO-2HA to generate C-terminal 2xHA fusions (Table 1). Each construct was introduced into wild-type EPEC, the ATPase null mutant Δ*sctN*, and in the SP deficient strains Δ*sctK*, Δ*sctL*, and Δ*sctQ* (Table 2). We confirmed that SP null mutants are nonpolar by restoring T3 secretion through plasmid-based expression of the respective proteins (Fig. S2). Then, T3-dependent secretion of the overproduced 2HA-tagged proteins was assessed. The SctI-HA protein was detected in the supernatant of wild-type EPEC, but not in the negative control Δ*sctN* (Fig. 1A). In contrast to previous observations for middle and late substrates (12), the early substrate SctI-HA was not detected in the supernatant of strains lacking SctK, SctL, or SctQ (Fig. 1A, panel SN). The protein levels of SctI-HA in total cell extracts were similar in these mutant strains, ruling out defects in protein expression (Fig. 1A, panel *P*).

**TABLE 1:** Plasmids used in this study

Name	Description^*a*^	Source or reference
pKD46	λ-Red recombinase system plasmid with an inducible *araB* promoter; AMP^r^	(48)
pKD4	Template plasmid used for amplification of the kanamycin resistance cassette used for the λ-Red recombinase system; AMP^r^ KAN^r^	(48)
pKD13	Template plasmid used for amplification of the kanamycin resistance cassette used for the λ-Red recombinase system; AMP^r^ KAN^r^	(48)
pFLP2	Plasmid used for expression of the Flp recombinase system; AMP^r^	(49)
pSUB11	Template plasmid used for amplification of the 3xFLAG epitope used for the λ-Red recombinase system; AMP^r^	(50)
pRE112	Suicide plasmid used for allelic exchange; CHL^r^	(51)
pRE_Δ*sctW*	pRE112 carrying an in-frame *sctW* deletion; CHL^r^	Gift of the Puente JL Lab
PCR-Blunt-II TOPO	Cloning vector; KAN^r^	Invitrogen
pARorf5BS	PCR-Blunt-II TOPO carrying *sctL*; KAN^r^	This study
pQE30	Plasmid used for expression of 6xHis-tagged proteins under the T5 promoter; AMP^r^	QIAGEN
pAQorf5	pQE30 encoding His-SctL; AMP^r^	This study
pTrc99A	Expression vector with the *trc* promoter; AMP^r^	Amersham-Pharmacia
pAT_*sctL*	pTrc99A carrying *sctL*; Ap^r^	This study
pTrc99A_FF4	Modified pTrc99A expression vector; AMP^r^	(52)
pMTH*sctK*	pTrc99AFF4 encoding His-SctK; AMP^r^	This study
pIT_*sctQ*	pTrc99AFF4 carrying *sctQ*; AMP^r^	This study
pST_*sctI*	pTrc99AFF4 carrying *sctI*; AMP^r^	This study
pBT_*sctF*	pTrc99AFF4 carrying *sctF*; AMP^r^	This study
pFT_*map*	pTrc99AFF4 carrying *map*; AMP^r^	This study
pET19b	Plasmid used for expression of 10xHis-tagged proteins under the T7 promoter; AMP^r^	Novagen
pJE_*sctI*	pET19b encoding His-SctI; AMP^r^	(26)
pNE_*sctF*	pET19b encoding His-SctF; AMP^r^	This study
pNE_*map*	pET19b encoding His-Map; AMP^r^	This study
pTOPO-2HA	pCR2.1-TOPO derivative carrying *Citrobacter rodentium espG* coding region tagged with two HA epitopes at the C-terminus; KAN^r^ AMP^r^	(53)
pJH_*map*	pTOPO-2HA carrying *map* with its native RBS; KAN^r^ AMP^r^	(26)
pSH_*sctA*	pTOPO-2HA carrying *sctA* with its native RBS; KAN^r^ AMP^r^	(12)
pJH_*sctI*	pTOPO-2HA carrying *sctI* with its native RBS; KAN^r^ AMP^r^	(26)
pJH_*sctF*	pTOPO-2HA carrying *sctF* with its native RBS; KAN^r^ AMP^r^	This study
pBADMycHisA	Plasmid used for expression of c-Myc and 6xHis-tagged proteins under the *araBAD* promoter; AMP^r^	Thermo Fisher
pVB_*sctK*	pBADMycHisA encoding SctK-His c-Myc; AMP^r^	This study
pNB_*sctI*	pBADMycHisA encoding SctI-His c-Myc; AMP^r^	This study

^*a*^ AMP^r^: ampicillin resistance; KAN^r^: kanamycin resistance; CHL^r^: chloramphenicol resistance.

**TABLE 2:** Strains used in this study

Strain	Description^*a*^	Source or reference
TOP10	Strain used for cloning; STR^r^	Invitrogen
XL1Blue	Strain used for plasmid propagation and DNA purification; TET^r^	Stratagene
SM10 λpir	Strain used to mobilize mobRP4 plasmids and for propagation of suicide plasmids containing R6K origin; KAN^r^	(54)
E2348/69	EPEC wild-type strain E2348/69 serovar O127:H6; STR^r^	(55)
Δ*sctN*	E2348/69 carrying an in-frame deletion of *sctN*; STR^r^	(56)
Δ*sctK*	E2348/69 carrying an *sctK* deletion; STR^r^ KAN^r^	(12)
Δ*sctQ*	E2348/69 carrying an *sctQ* deletion; STR^r^ KAN^r^	(12)
Δ*sctL*	E2348/69 carrying an *sctL* deletion; STR^r^ KAN^r^	(12)
Δ*sctW*	E2348/69 carrying an in-frame deletion of *sctW*; STR^r^	Gift of the Puente JL Lab
Δ*cesL*	E2348/69 carrying a *cesL* deletion; STR^r^ KAN^r^	(30)
Δ*sepD*	E2348/69 carrying an in-frame deletion of *sepD*; STR^r^	Gift of the Puente JL Lab
Δ*sctF*	E2348/69 carrying an *sctF* deletion; STR^r^ KAN^r^	This study
Δ*sctI*	E2348/69 carrying an *sctI* deletion; STR^r^ KAN^r^	This study
Δ*espC*	E2348/69 carrying an in-frame deletion of *espC*; STR^r^	Gift of the Navarro-Garcia F Lab
Δ*sctK*Δ*sctW*	E2348/69 carrying an *sctK* and in-frame *sctW* deletions; STR^r^ KAN^r^	This study
Δ*sctK*Δ*cesL*	E2348/69 carrying an *sctK* and *cesL* deletions; STR^r^ KAN^r^	This study
Δ*sctK*Δ*sepD*	E2348/69 carrying an *sctK* and in-frame *sepD* deletions; STR^r^ KAN^r^	This study
Δ*sctL*Δ*sctW*	E2348/69 carrying an *sctL* and in-frame *sctW* deletions; STR^r^ KAN^r^	This study
Δ*sctQ*Δ*sctW*	E2348/69 carrying an *sctQ* and in-frame *sctW* deletions; STR^r^ KAN^r^	This study
Δ*sctF*Δ*sctK*	E2348/69 carrying an *sctK* and *sctF* deletions; STR^r^ KAN^r^	This study
Δ*sctF*Δ*sctL*	E2348/69 carrying an *sctL* and *sctF* deletions; STR^r^ KAN^r^	This study
Δ*espC* Δ*sctK*	E2348/69 carrying an in-frame *espC* and *sctK* deletions; STR^r^ KAN^r^	This study
*sctW*-3FLAG Δ*sctK*	E2348/69 carrying an *sctK* deletion and expressing 3xFLAG-tagged SctW; STR^r^ KAN^r^	This study
*grlA*-3FLAG Δ*sctK*	E2348/69 carrying an *sctK* deletion and expressing 3xFLAG-tagged GrlA; STR^r^ KAN^r^	This study
*cesL*-3FLAG Δ*sctK*	E2348/69 carrying an *sctK* deletion and expressing 3xFLAG-tagged CesL; STR^r^ KAN^r^	This study
*sctW*-3FLAG Δ*sctQ* Δ*sctK*	E2348/69 carrying an *sctK* and *sctQ* deletions and expressing 3xFLAG-tagged SctW; STR^r^ KAN^r^	This study
Δ*sctC*	E2348/69 carrying an in-frame deletion of *sctC*; STR^r^	Gift of the Puente JL Lab
Δ*sctC* Δ*sctK*	E2348/69 carrying an *sctK* and *sctC* deletions; STR^r^ KAN^r^	This study

^*a*^ STR^r^: streptomycin resistance; KAN^r^: kanamycin resistance; TET^r^: tetracycline resistance.

Next, we sought to examine the secretion pattern of SctF, but the HA-tagged version was undetectable, likely due to instability. To address this issue, we expressed untagged SctF from the pTrc99A_FF4 plasmid and confirmed its presence, using polyclonal antibodies, in the supernatant of wild-type EPEC and the Δ*sctF* mutant strain (Fig. 1B, panel SN). Importantly, untagged substrates, such as Map, expressed from plasmid pTrc99A_FF4 recapitulated the bypass effect in SP-deficient mutants (Fig. S3). However, in accordance with SctI-HA, SctF overproduction did not bypass the need for the sorting platform, confirming its essential role in early substrate secretion (Fig. 1B). Overall, the inability of early substrates to circumvent SP deficiency, even when overproduced, indicates that the components assembling the SP structure are entirely essential for secreting this category of substrates.

### The SctI needle adapter protein and the SctF needle protein are essential for the secretion of overproduced substrates

**Fig 2:**
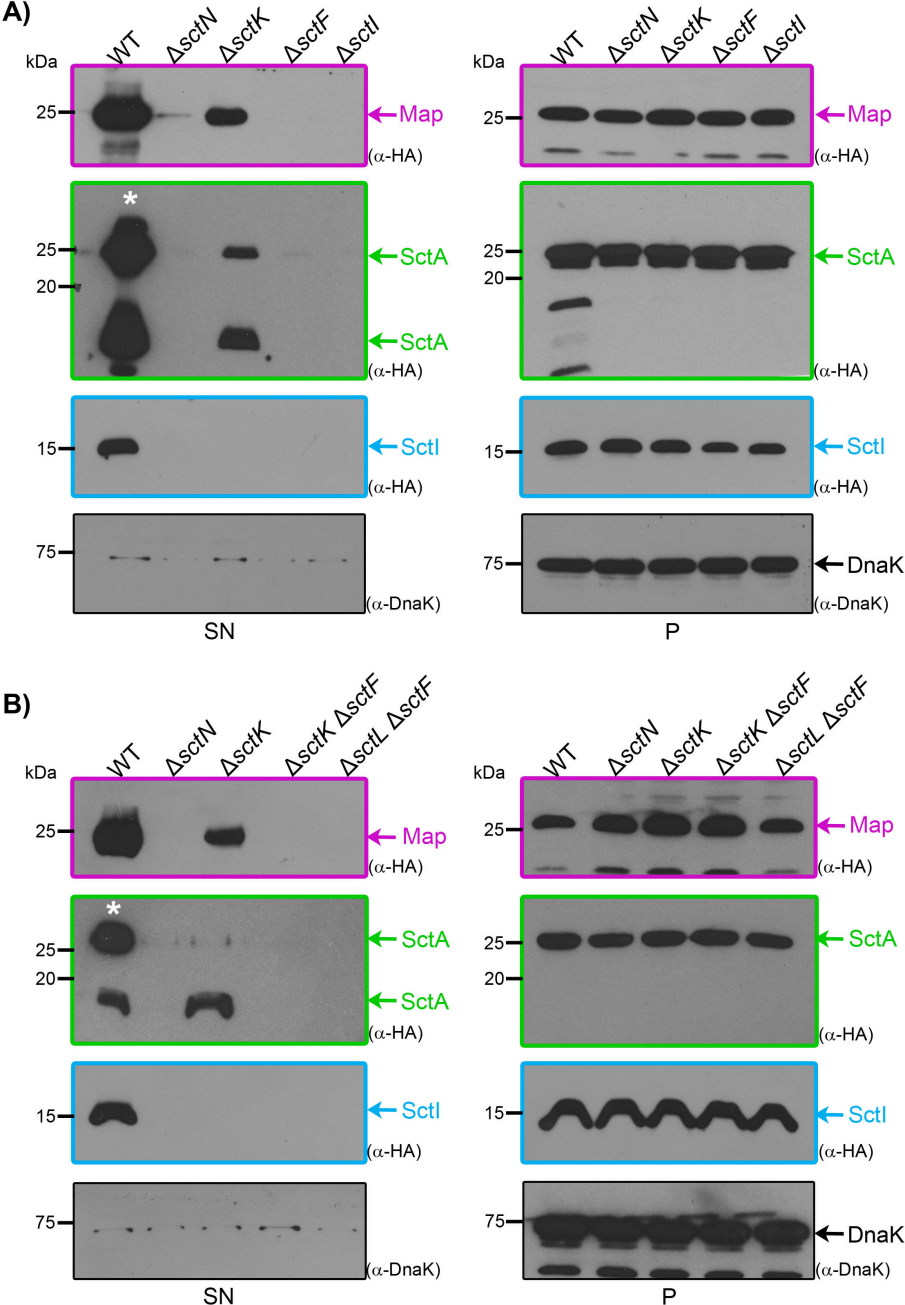
The needle SctF and the inner rod SctI proteins are essential for type III secretion of overproduced substrates in EPEC. (**A and B**) Wild-type EPEC (WT) or the indicated nonpolar mutant strains carrying a high-copy number plasmid (pTOPO2HA) expressing C-terminally HA-tagged early (SctI), middle (SctA), or late (Map) type III secretion substrates were cultured under T3SS-inducing conditions. The presence of each substrate in the culture supernatant (SN) or whole-cell lysate (**P**) was assessed by immunoblotting with anti-HA antibodies. The cytosolic chaperone DnaK served as a loading control for bacterial whole-cell lysates and as a cell lysis control in the bacterial supernatant. To prevent signal saturation, the amount of the SctA-HA supernatant sample loaded in the WT strain (*) was half of that of the other samples. Results are representative of three independent biological replicates. Molecular mass markers are shown on the left (kDa). Substrates are color-coded in magenta (late substrate), green (middle substrate), and blue (early substrate).

While formation of the extracellular needle structure is not necessary for assembling either the membrane-embedded basal body (57, 58) or the SP (59), needle assembly has been proposed to play a role in opening the periplasmic gate of the SctC secretin ring (60). In addition, it has been reported that in the absence of the SP components, the needle is not assembled (39–41, 44, 45). However, our prior and current results (see below) demonstrate that, when overproduced, middle and late substrates are secreted even in mutants lacking SP components (12). To further investigate the role of the SctI needle adapter and the SctF needle under conditions of T3 substrate overproduction, we examined the secretion profile of SctI-HA (early), SctA-HA (middle), and Map-HA (late) substrates in strains lacking either the needle adapter protein SctI or the needle protein SctF. The three substrates were produced at equivalent levels in all tested strains and were readily detected in the supernatant of wild-type EPEC, but not in the negative control Δ*sctN* mutant (Fig. 2A). In line with our previous observations (12) and (Fig. 1A), SctA-HA and Map-HA were consistently detected in the supernatant of the Δ*sctK* mutant strain, whereas SctI-HA was not. However, overexpression of these substrates failed to compensate for the absence of either the needle adapter SctI or the needle SctF proteins (Fig. 2A panel SN). It is important to mention that the SctI-HA protein was not secreted in the Δ*sctI* mutant strain, most likely due to steric hindrance caused by the HA tag, which may have interfered with the proper assembly of the needle adapter, and consequently, with its ability to complement protein secretion (Fig. S4). Notably, when analyzing SctA-HA secretion, we observed additional lower-molecular mass bands alongside the expected 25 kDa band of full-length SctA. Since SctA is a known target of the serine protease autotransporter EspC (61), we hypothesized that these lower-molecular weight species might result from EspC proteolytic activity. Supporting this, deletion of *espC* from both wild-type EPEC and *sctK* mutant strains led to the exclusive detection of full-length SctA (Fig. S5). These results emphasize the crucial role of the needle adapter and the needle proteins in type III secretion of overproduced substrates.

Our results confirmed that overproduced middle and late T3 substrates are secreted in the absence of SctK (Fig. 2A), indicating that a functional secretion channel must remain even without an intact cytosolic SP. Given that an open-gate conformation of the outer membrane secretin is required for substrate passage through the T3SS (60), we hypothesized that the bypass effect observed for middle/late substrates might also depend on the structural integrity of this protein. Supporting this, deletion of the secretin-encoding gene *sctC* completely abolished the ability of the overproduced late substrate Map to bypass the absence of the SP component SctK (Fig. S6). To further investigate whether the needle protein is required for substrate secretion in the absence of the SP, we examined the secretion of overproduced substrates by the double null mutants Δ*sctK* Δ*sctF* and Δ*sctL* Δ*sctF* (Fig. 2B). We found that the absence of SctF completely abrogates substrate secretion in both Δ*sctK* and Δ*sctL* strains. These results indicate that the bypass effect observed in the absence of the SP proteins SctK and SctL depends on the SctF needle protein to facilitate T3 secretion.

### Cooperative interplay between the sorting platform and the gatekeeper complex for substrate recruitment

The finding that middle and late substrates, but not early substrates, bypass the secretion defect in SP-deficient mutants (Fig. 2) led us to explore a functional link between the SP and substrate hierarchy regulation. In EPEC, SctW forms a tight complex with the SepD and CesL proteins, which binds to the cytosolic portion of SctV at the export gate. This trimeric complex acts as a molecular switch, facilitating the release of middle substrates while preventing premature secretion of late substrates before host cell contact (30–33, 62). Deletion of any component of the SctW complex disrupts this regulation, resulting in abolished secretion of middle substrates and uncontrolled hypersecretion of late substrates (30–32).

**Fig 3:**
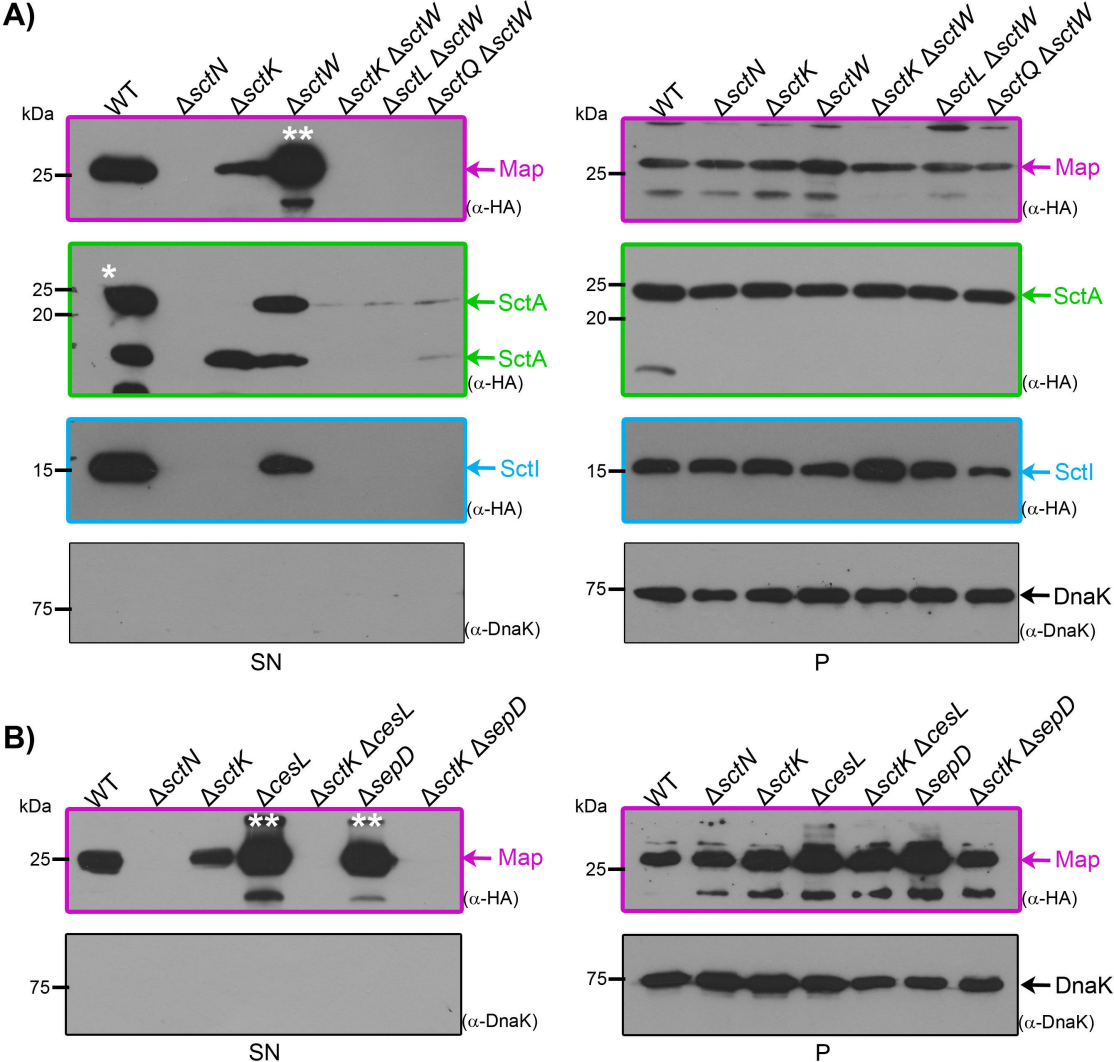
Cooperative effect of the sorting platform and the gatekeeper complex on type III substrate recognition. (**A and B**) Wild-type EPEC (WT) or the specified single or double mutant strains were cultured under T3SS-inducing conditions. All strains carry a pTOPO-2HA plasmid expressing C-terminally HA-tagged early (SctI), middle (SctA), or late (Map) type III secretion substrate. The presence of the indicated substrate in the culture supernatant (SN) or in the whole-cell lysate (**P**) was assessed by immunoblotting with anti-HA antibodies. To prevent signal saturation, the volume of the supernatant sample of Map-HA in strains Δ*sctW*, Δ*sepD,* and Δ*cesL* (**) was reduced to one-third of the volume loaded for the other samples. Likewise, the amount of the SctA-HA supernatant sample loaded in the WT strain (*) was half of that of the other samples. The cytosolic chaperone DnaK was used as a loading control for bacterial whole-cell lysates (**P**) and as a cell lysis control in the bacterial supernatant (SN). Results are representative of three independent biological replicates. Molecular mass markers are shown on the left (kDa). Substrates are color-coded in magenta (late substrate), in green (middle substrate), and in blue (early substrate).

To gain further insights into the molecular mechanisms that control the hierarchical recruitment of substrates into the T3S machinery, we compared the secretion profile of distinct categories of overproduced substrates in single- and double-deletion mutants of the SctW complex and SP components. Deletion of *sctW* resulted in hypersecretion of the late substrate Map-HA, confirming its expected behavior in a gatekeeper-deficient mutant (Fig. 3A, panel SN). Notably, though at reduced levels, the middle substrate SctA-HA was also secreted in this mutant background, indicating that overproduced SctA-HA can be recognized by the SP and by SctV, bypassing the need for SctW. Furthermore, we found that SctW contributes to early substrate secretion, as reduced levels of SctI-HA were detected in the supernatant of the Δ*sctW* strain compared to wild-type EPEC (Fig. 3A, panel SN).

Remarkably, the secretion of all categories of T3 substrates was completely abolished in the Δ*sctK* Δ*sctW*, Δ*sctL* Δ*sctW,* and Δ*sctQ* Δ*sctW* double mutants. The lack of secretion of the late substrate Map-HA in these strains is particularly striking as it contrasts with the secretion levels observed, for example, in the single Δ*sctK* and Δ*sctW* deletion mutants (Fig. 3A). These results indicate that the bypass effect seen in SP-deficient mutants upon overproduction of middle and late substrates relies on SctW, revealing a functional interplay between the SP and the gatekeeper SctW protein.

To investigate the functional relationship between the gatekeeper complex and the SP in greater depth, we examined the secretion of the overproduced late substrate Map-HA in Δ*sctK* Δ*cesL* and Δ*sctK* Δ*sepD* double mutants (Fig. 3B). Consistent with our observations in the Δ*sctK* Δ*sctW* double mutant, Map-HA secretion was also completely abolished (Fig. 3B). These results suggest that the SP and the entire gatekeeper complex work in a cooperative manner to efficiently recruit cytosolic T3S substrates to the vicinity of the export gate for their secretion.

### The gatekeeper complex forms a protein assembly with the sorting platform component SctK

**Fig 4:**
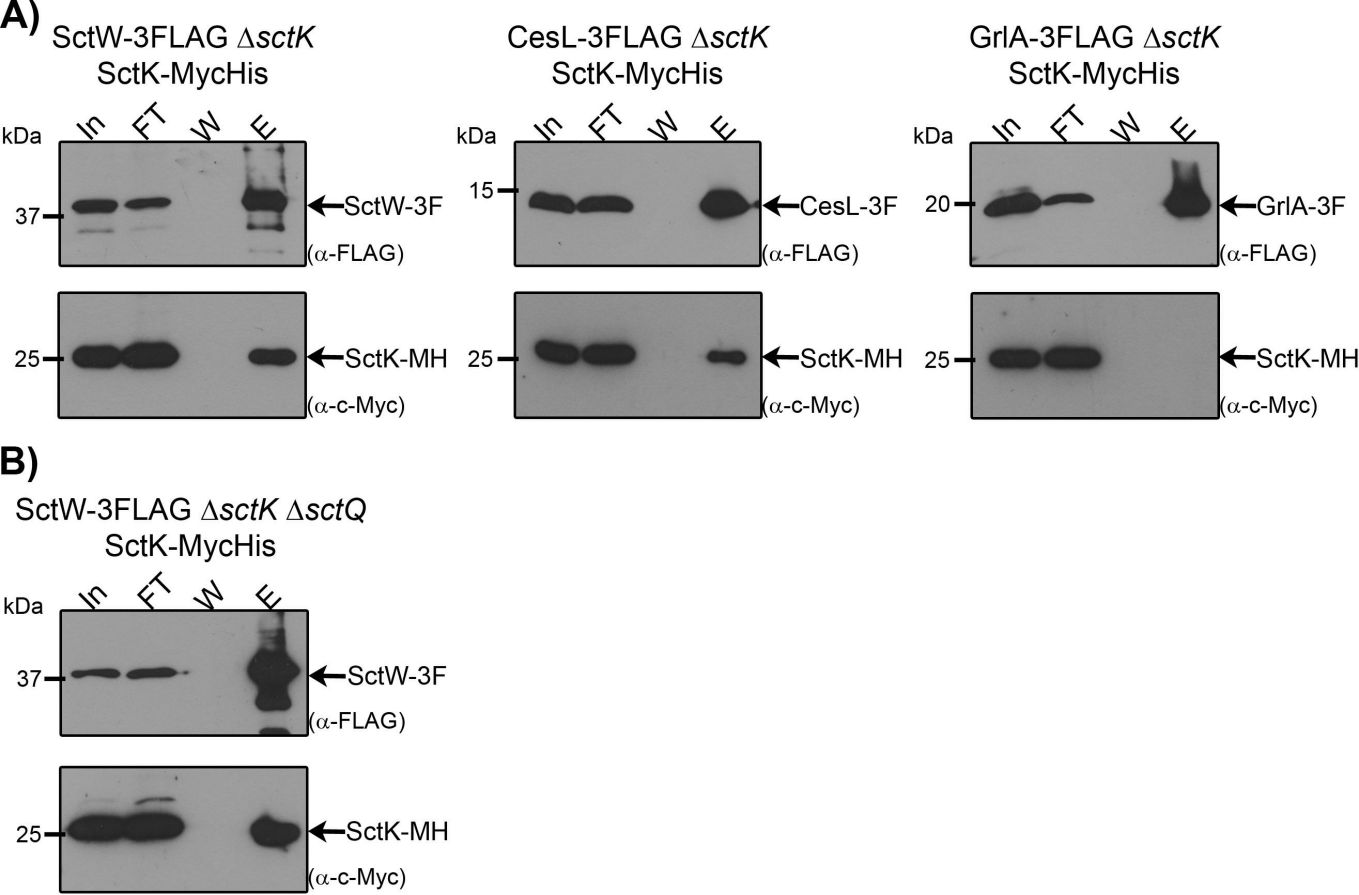
SctK forms a complex with the gatekeeper complex SctW/SepD/CesL. (**A**) Δ*sctK* strains chromosomally encoding either SctW-3FLAG, CesL-3FLAG, or GrlA-3FLAG and harboring a plasmid expressing SctK-Myc-His (MH) were cultured under T3SS-inducing conditions. Cleared whole-cell lysates were used as input (In) for immunoprecipitation using anti-FLAG resin. The flowthrough (FT) was discarded, and after extensive washing (**W**), bound proteins were eluted (**E**). Resulting fractions were analyzed by immunodetection probing for SctK (with anti-c-Myc antibodies) and for SctW, CesL, or GrlA (with anti-FLAG antibodies). (**B**) A Δ*sctK* Δ*sctQ* double-mutant strain chromosomally encoding SctW-3FLAG and harboring a plasmid expressing SctK-MH was used for a co-immunoprecipitation experiment in the same conditions as described in (**A**). Results are representative of three independent biological replicates.

The functional relationship between the SP and the gatekeeper complex prompted us to investigate the potential interaction between SctK and the SctW/SepD/CesL complex. We constructed EPEC Δ*sctK* derivatives chromosomally expressing 3xFLAG-tagged SctW or CesL and expressing MycHis-tagged SctK *in trans* from an arabinose-inducible plasmid to conduct co-immunoprecipitation (co-IP) assays. Importantly, we previously reported that the C-terminal 3xFLAG tag did not affect the function of either SctW or CesL (30, 31); conversely, epitope tagging at the C-terminus of SepD impairs its proper function (31), thereby preventing us from testing the SepD-SctK co-IP. The complemented Δ*sctK* strain expressing a chromosomal 3xFLAG-tagged version of GrlA, a transcription factor that is not a structural component of the injectisome (53, 63), served as a negative control for the co-IP assays. Co-IP results showed that SctK-MycHis co-eluted specifically with both SctW-3FLAG and CesL-3FLAG, but not with the negative control GrlA-3FLAG (Fig. 4A), indicating that SctK forms a complex with components of the gatekeeper complex *in vivo*.

However, our co-IP results do not rule out the possibility of an indirect interaction between these proteins, requiring additional injectisome components to bridge SctK and the gatekeeper complex. Given that SctQ is the major component of the SP pods and scaffolds the entire SP by directly interacting with SctK (12, 43, 64), this raises the possibility that SctQ may mediate the interaction between SctK and SctW. To test this, we introduced a Δ*sctQ* deletion into the SctW-3FLAG Δ*sctK* strain and performed the co-IP assay to examine the interaction between SctK and SctW in the absence of SctQ. As shown in Fig. 4B, SctK-MycHis co-immunoprecipitates with SctW-3FLAG at similar levels, indicating that SctQ is not required for the association between SctK and SctW.

## DISCUSSION

The T3SS is one of the most complex bacterial machineries known to date. Its assembly and proper functioning rely on the sequential secretion of proteins, classified as early, middle, and late substrates, each recognized at specific stages of injectisome assembly. To achieve this timely secretion, multiple cytosolic components act in concert, ensuring the opportune delivery of substrates. At the center of this process lies the sorting platform, which is posited to serve as a physical scaffold where the distinct categories of substrates are sequentially loaded and then initiated into the secretion pathway. Despite its central role for secretion hierarchy, the precise mechanisms governing SP function remain unclear. In this study, we aimed to shed light on the role of the SP in the hierarchical substrate secretion process.

To gain insights into how the SP participates in recognizing distinct substrate categories, we leveraged our previously established secretion assay employing overexpressed substrates (12). This approach allowed us to genetically identify injectisome components involved in directing substrates to the export gate. Our findings revealed a contrasting secretion pattern between early and middle/late substrates. While overproduction of middle and late substrates compensated for the absence of either SctK or SctL, early substrate secretion strictly required all SP components (Fig. 1). These results suggest that the SP acts as a critical binding hub for early substrates, ensuring their timely secretion. A similar observation has been reported in *Shigella,* where both SctK and SctL are needed for the transit of SctI and SctF through the T3SS but are dispensable for middle and late substrates (44). However, in contrast to our results, the overexpression of SctI or SctF in the *sctK* and *sctL* SP mutants, respectively, compensates for the absence of the SP component, allowing early substrate secretion and the assembly of the T3SS (44). Both findings underscore the relevance of SP components in early substrate recognition, with differences likely attributable to species-specific molecular mechanisms across T3SS families.

Although there are conflicting reports about the interaction of SctI and SctF with SctQ (45, 65), evidence supports these interactions (22, 65), as well as interactions between SP components and the export gate proteins SctU and SctV (22, 66), suggesting that SP components provide essential docking sites for conveying early substrates to the export gate. Further studies will be needed to elucidate the molecular mechanisms by which SP components ensure the timely recognition and delivery of early substrates.

It has been shown that mutants lacking SP proteins fail to assemble the extracellular needle structure (44, 45). More recently, cryo-electron tomography studies using minicells of *Shigella* and *Salmonella* have provided high-resolution *in situ* structures of the T3SS, confirming that the needle structure is not assembled in the absence of SP components (39–41). Here, we found that both the needle adapter SctI and needle SctF proteins are essential for the secretion of all substrate categories as their absence cannot be bypassed by overexpression of either middle or late substrates (Fig. 2). Therefore, an intriguing question arises: How do middle and late substrates transit through the secretion channel when the SP is compromised? Our findings suggest that both the needle adapter and needle proteins may have a cytoplasmic role needed for recognition and secretion of the next tier of substrates. In agreement with this hypothesis, it has been reported that SctI interacts with SctW, an interaction proposed to regulate the secretion of middle and late substrates (67, 68). Moreover, an SctI–SctU interaction has also been shown and implicated in the correct regulation of late substrate secretion (20, 68, 69). Besides, works by us and others have demonstrated that both SctI and SctF interact with SctP (26, 69), somehow implicating these proteins in the first substrate specificity switching mechanism. In addition, it has been reported that single alanine mutations in SctI are deficient in substrate specificity switching, showing impaired secretion of a middle substrate (19, 70). Remarkably, it was demonstrated that these mutations did not affect inner rod assembly, suggesting a further cytoplasmic role of the SctI protein (19). Furthermore, by means of cryo-EM visualization of the *Salmonella* T3SS needle complex, a mechanism for substrate-mediated gate opening of the outer membrane secretin has been hypothesized, in which early substrates facilitate an initial allosteric transition to unlock the gate (60). Although speculative at this point, under the experimental conditions used in this study, overproduced middle and late substrates might also trigger the conformational changes associated with the secretin gate opening for their secretion. Additional experimentation will be needed to fully address this hypothesis.

The SctW/SepD/CesL complex plays a crucial role in regulating the secretion hierarchy by interacting with the export gate proteins SctU and SctV. Its interaction with SctV creates a docking site for middle substrates, facilitating the orderly transit of translocators prior to late substrate secretion, thereby ensuring the assembly of the translocation pore into the host cell membrane before the release of late substrates (30–32, 62). Since null mutants in any component of the SctW complex result in hypersecretion of effectors, we reasoned that deleting *sctW* would exacerbate the bypass secretion phenotype observed in the Δ*sctK* and Δ*sctL* single mutants when late substrates are overproduced. However, unexpectedly, in double deletion strains Δ*sctK* Δ*sctW* and Δ*sctL* Δ*sctW*, late substrates were no longer recognized by the T3 secretion machinery (Fig. 3). This finding suggests that late substrates are cooperatively recognized by these two interacting sites and highlights the interplay between the SP and the SctW complex in handing over cytosolic effector/chaperone complexes into the SctV antechamber. Alternatively, since multiple export signals have been described in flagellar and virulence T3 substrates, which are recognized by different T3SS components such as chaperones or export gate proteins (71–75), it is tempting to speculate that the SP and the SctW complex might assist in secretion hierarchy through the recognition of distinct secretion signals.

Given the functional relationship that we found between the SP and the SctW molecular switch, we sought to investigate the potential physical interaction between these two cytosolic complexes. Our Co-IP data showed that SctK associates with the SctW complex, co-immunoprecipitating with both SctW and CesL (Fig. 4A); nevertheless, these protein interactions might be facilitated by additional cytosolic components. However, we found that the SctK–SctW interaction is not mediated by SctQ as SctK co-immunoprecipitates with SctW in a Δ*sctQ* mutant (Fig. 4B). Given that the established interaction chain for cytosolic components follows the sequence SctK-StcQ-SctL-SctN-SctO and considering the proposed pathway for assembly of the SP (43, 76), the fact that SctK associates with SctW in the absence of SctQ suggests that this interaction is likely mediated neither by SctL nor by the ATPase complex components, SctN and SctO. Interestingly, previous reports have identified an interaction between SctW and the SP proteins SctQ and SctL (65, 77), indicating that SctW may require binding to the SP to fulfill its multiple regulatory roles in substrate hierarchy. As the gatekeeper SctW protein is required for substrate loading onto the SP (13), it is plausible that the captured complex reflects the handover of substrates from the SctW complex to the SP scaffold. In accordance with this hypothesis, interactions of SctW with a middle substrate chaperone have been reported (67, 78). Another possibility is that SctK assists in positioning the SctW complex near the export gate and the ATPase complex. In any case, our results suggest that SctK is not merely functioning as a symmetry adapter protein between the basal body SctD ring and SctQ but is also playing an active role, either as a soluble or assembled component, in the protein–protein interaction framework needed for proper T3SS assembly. Further investigation will be necessary to determine the functional implications of this novel interaction. Taken together, our findings suggest that both the SP and the SctW complex participate in a dynamic network of protein interactions that establishes the recognition and sorting mechanisms of type III substrates.

In addition to its well-established role in secretion switching from middle to late substrates, a recent study suggested that the SctW complex also enhances the affinity of SctN for early substrate–chaperone complexes (79). In agreement, we found reduced secretion levels of overproduced early substrate SctI in the absence of SctW (Fig. 3A). To the best of our knowledge, this is the first report directly showing that the absence of the SctW complex negatively affects early substrate secretion. However, the possibility that the reduced secretion of SctI could result from competition at the export gate, due to late substrates being hypersecreted in the Δ*sctW* background and potentially saturating the gate, should be addressed in future investigations. In any case, the underlying functional significance of this phenotype remains to be further investigated.

It has been proposed that the SP SctQ and SctL proteins exhibit a dynamic behavior, continuously exchanging between the cage-like assembled state at the base of the injectisome and free subcomplexes in the cytoplasm (46, 47, 80). Although the functional role of such dynamic cycles is not quite clear yet, they appear to occur in response to environmental cues, ensuring that the T3SS actively secretes late substrates only when the bacterium is in intimate contact with the target host cell. Further, it was shown that the binding of SctQ to effectors functions as a substrate shuttle to the export gate (47), an observation consistent with the essential role of SctQ in late substrate secretion (12, 13). This proposed mechanism is similar to the one reported in the flagellar system, where a cytosolic pool of FliH (SctL) in complex with the ATPase FliI (SctN) acts as a dynamic carrier for flagellar substrates (81). Moreover, in line with the indispensable role of the SP for early substrate secretion described in this study, interactions of SctQ with early substrates have been detected *in vitro* (22, 65); hence, it is possible to hypothesize that the SctQ protein carrier function could be determined by different protein interaction affinities, which allows a differential recruitment of the three categories of substrates to the base of the injectisome.

**Fig 5:**
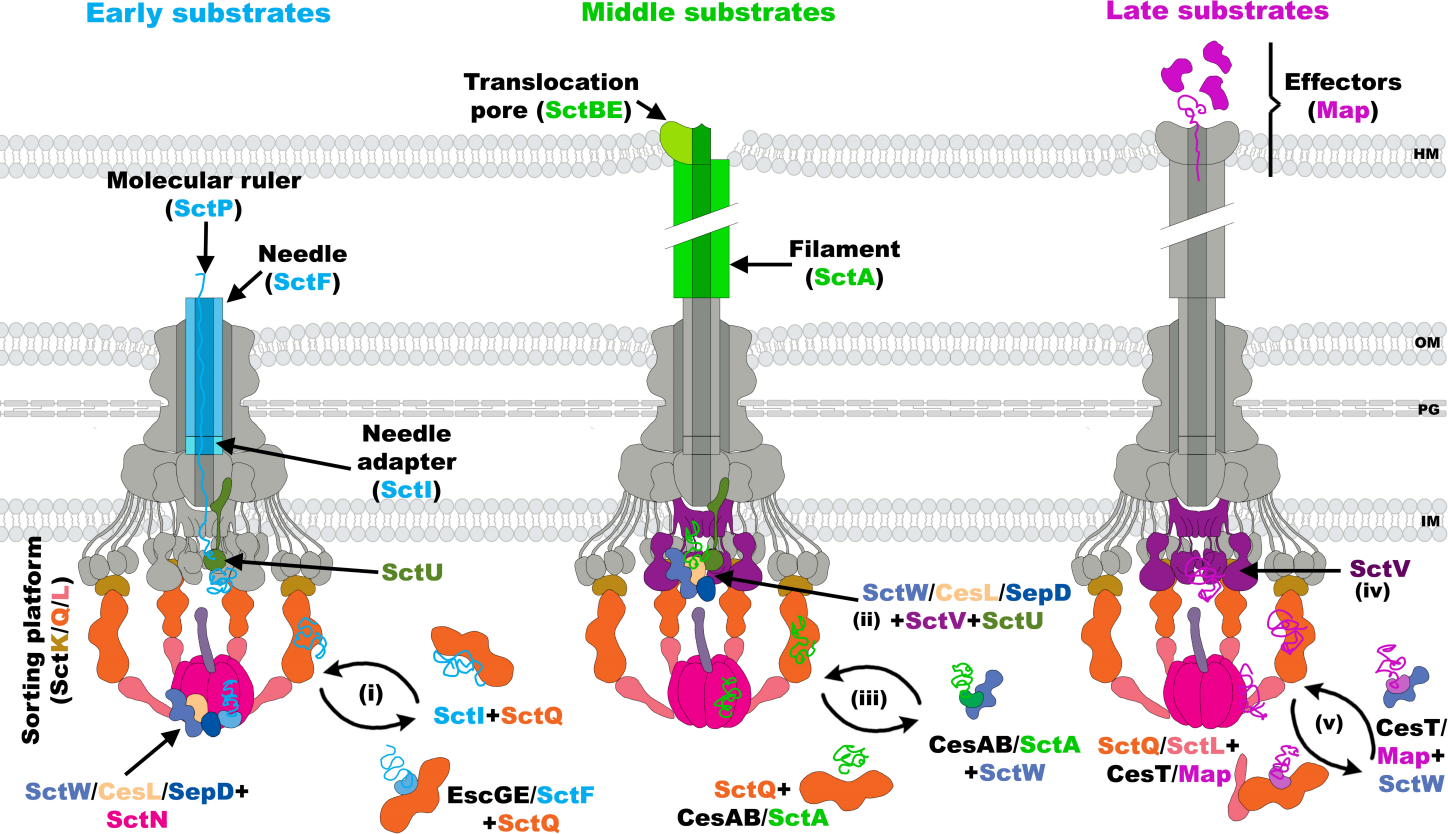
Model for sorting platform hierarchical recognition of T3SS substrates. The three stages of type III protein hierarchical secretion are shown, color-coded: early (blue), middle (green), and late (magenta) substrates. Once the sorting platform has been assembled onto the needle-complex, (**I**) cytosolic SP proteins or subcomplexes, represented here by SctQ, may recognize and shuttle early substrates as SctI, either alone or in complex with its chaperones SctF/EscGE to the T3SS base. Early substrates are then recognized by the export gate protein SctU and secreted for its subsequent assembly. After completion of needle assembly, SctP binding to SctU stops early substrate recognition. (ii) The SctW complex reaches the export gate components forming a high-affinity recognition platform for middle substrates. The middle substrates are then delivered to the SctW/CesL/SepD + SctU/SctV platform by either (iii) SctW or the SP shuttling function (iv) Finally, assembly of the translocation pore into the host cell membrane allows late substrate recognition exerted by SctV. Late substrates might be recruited to the injectisome base either by (**V**) SctW binding to the chaperone CesT or to the SctQ/SctL complex.

Based on our results and insights from previous studies, we propose the following model for the SP–gatekeeper complex interplay for substrate recognition and sorting (Fig. 5). Upon assembly of the basal body and export apparatus: (i) the cytosolic SP components (exemplified by SctQ) could be preloaded with early substrates SctI/SctF or chaperone–early substrate complexes (EscGE/SctF) and recruited to the base of the injectisome (22, 65). The SctW gatekeeper complex could then be positioned inside the SP enclosure through its interaction with SctK or SctQ, where it would cooperate with SctN to facilitate the secretion of early substrates through the export gate (65, 79). Once the extracellular needle reaches its full length, the first substrate specificity switch occurs (26–29). At this stage, (ii) the SctW gatekeeper complex interacts with the export gate components SctU and SctV (30–32, 82), where it forms a high-affinity recognition platform for middle substrates, while simultaneously blocking the recognition of late substrates (32). (iii) The SP and the gatekeeper complex may cooperatively increase the local concentration of middle substrates, facilitating their secretion (78, 83). Upon completion of the translocation pore in the host cell membrane, a yet-to-be fully described signal is transmitted to the export gate, (iv) relieving the repression of late substrate recognition exerted by the SctW complex on SctV (31, 32, 34–36, 38). SctV can then recognize late substrate–chaperone complexes with high affinity (31, 32, 36, 38). These effector complexes may be transported by (v) SP components or subcomplexes (exemplified by the SctQ-SctL complex) (47) or by SctW to the SP (77). For this model to function effectively, the substrate shuttling mechanism must depend on differential binding affinities between substrate–chaperone complexes and the SP components. It is thus tempting to hypothesize that SP components or soluble complexes exhibit different affinities for distinct substrate categories, possibly enabling a mechanism for differential recognition of substrates.

Overall, our data shed light on the yet-to-be-fully characterized mechanisms of T3SS assembly and highlight the cooperative interplay between the sorting platform and the gatekeeper complex, two major players implicated in substrate recognition and secretion hierarchy. These findings pave the way for a deeper understanding of the molecular mechanisms governing the fine regulation required for T3SS assembly.

## MATERIALS AND METHODS

### Growth of bacterial strains

Bacterial strains used in this study are listed in Table 2. *E. coli* strains were routinely grown in lysogeny broth (LB) at 37°C and constant shaking at 250 rpm. For T3SS-inducing conditions, EPEC strains were grown in Dulbecco’s modified Eagle medium (DMEM; Gibco) at 37°C with a 5% CO_2_ atmosphere in static conditions. When necessary, antibiotics were added at the following concentrations: 100 µg/mL ampicillin; 50 µg/mL kanamycin; 50 µg/mL streptomycin; 25 µg/mL tetracycline; and 25 µg/mL chloramphenicol.

### Generation of EPEC null mutants and epitope-tagged strains

EPEC mutant strains were generated using either the Lambda Red (λ-Red) recombinase system or by *sacB*-based allelic exchange (48, 51) (Table 2). Plasmids and oligonucleotides used in this study are listed in Tables 1 and 3, respectively. Briefly, for the λ-Red system, a kanamycin resistance cassette was amplified from pKD4 or pKD13 vectors using oligonucleotides flanked by homologous sequences upstream and downstream of the target gene. The resulting PCR products were electroporated into the desired EPEC strain harboring the pKD46 vector. Mutant strains were subsequently selected on kanamycin plates and verified by colony PCR. When required, the kanamycin cassette was excised from the mutant strain employing the plasmid pFLP2, which expresses the FLP recombinase. To generate double-mutant strains, the second mutation was introduced following the same procedure. Alternatively, for the *sacB*-based allelic exchange method, an in-frame deletion was amplified from EPEC chromosomal DNA by nested PCR. The resulting PCR product was cloned into the suicide plasmid pRE112 and introduced into *E. coli* SM10 λpir. Conjugation with the target EPEC strain was performed, and transconjugants were selected on chloramphenicol plates. Following sucrose counter-selection, successful allelic exchange was confirmed by sensitivity to chloramphenicol and resistance to sucrose and further confirmed by colony PCR.

**TABLE 3:** Oligonucleotides designed in this study

Oligonucleotide	Sequence 5’–3’
sctK-NcoIF	GAAAAATACCATGGCAATTTTTAATAA
sctK-H3R	TTTAAAGCTTTAAAGTTTCATAAGGC
mutsctFFw	ATTATTTTATTAACTTCTGAGGGAAATTTAATGAATTTATCTGAAATTACGTGTAGGCTGGAGCTGCTTC
mutsctFRv	TATCATTAACCATAAAAATTAAAAACTACGGTTAGAAATGGTTGAGACCAATGAATATCCTCCTTAGTTC
mutsctIFw	GCATCGAAACTAATTTTGTAACCGATGTTGAAAGGAGGCTTGCTAACTTAGTGTAGGCTGGAGCTGCTTC
mutsctIRv	TCGCATCGCATAAAAATAGAGAGGTAATGGATGCATTATGCTATTGCCTCATTCCGGGGATCCGTCGACC
Gib_uni_pTrc_f	GGGGATCCTCTAGAGTCGACC
Gib_uni_pTrc_r	CATATGCTGTTTCCTGTGTGAAATTG
Gib_sctQ_pTrc_f	AATTTCACACAGGAAACAGCATATGAAGCCATTGAGTTCACAATTGA
Gib_sctQ_pTrc_r	GGTCGACTCTAGAGGATCCCCTTAATCACATACTACGCTAATA
sctINcoIFw	GAGAGGCCATGGATGCATTATGC
sctIHindIIIRv	TTTTTAAGCTTTTATTGCATCGAA
orf5BamF	CCAACTCGGATCCATGATTTATTTC
orf5SalR	GTCATTAATGTCGACATATCATCA
SctF-F	TGAGGGAAATCATATGAATTTATC
SctF-R	GCAGAAATATCAGGATCCATAAAA
SctFforHindIII	TTAAAAAAAGCTTATTTTATTAAC
SctFRevXhoI	GTAGTTTTCTCGAGTTATGGTTAA
MapF	GGTTTATCATATGTTTAGTCCAAC
MapR	AGTAAGTAAGCTTCTACAGCCGAG

To generate *sctF* and *sctI* null mutants, the kanamycin resistance cassette was amplified from pKD4 or pKD13 vectors using the oligonucleotide pair mutsctFFw/mutsctFRv or mutsctIFw/mutsctIRv, respectively. The resulting PCR products were electroporated into the WT EPEC strain harboring the pKD46 vector. Subsequently, to generate the double mutants Δ*sctF* Δ*sctL* and Δ*sctF* Δ*sctK*, the kanamycin cassette was excised from the Δ*sctF* strain, using the FLP recombinase encoded in plasmid pFLP2 (49). Then, deletion of either *sctK* or *sctL* was performed as previously reported (12). The strains Δ*espC* Δ*sctK,* Δ*sctC* Δ*sctK,* and Δ*sctK* Δ*sepD* were generated by deleting *sctK* in the corresponding genetic background Δ*espC,* Δ*sctC*, or Δ*sepD*, as reported previously (12). Construction of the Δ*sctK* Δ*cesL* double mutant was performed by excision of the kanamycin cassette from the *sctK* mutant, as described before (12), and the deletion in *cesL* was generated as previously reported (30). The double mutants Δ*sctK* Δ*sctW*, Δ*sctL* Δ*sctW*, and Δ*sctQ* Δ*sctW* were generated by introducing the suicide plasmid pRE_*sctW* into the Δ*sctK*, Δ*sctL*, and Δ*sctQ* strains by conjugation.

To generate *sctW*-3FLAG Δ*sctK* and *grlA*-3FLAG Δ*sctK* strains, the kanamycin cassette was excised from *sctW*-3FLAG and *grlA*-3FLAG strains, using the FLP recombinase, as mentioned above. Then, deletion of the *sctK* gene was performed as previously reported (12). In the case of the *cesL*-3FLAG Δ*sctK* strain, the *cesL* gene was tagged using a modified λ-Red recombinase system as reported in (50) and introduced into the Δ*sctK* strain harboring plasmid pKD46. The *sctW*-3FLAG Δ*sctQ* Δ*sctK* strain was generated by deleting *sctK* in the *sctW*-3FLAG Δ*sctQ* genetic background using the λ-Red recombinase system as previously reported (12).

### Generation of DNA constructs

Plasmids used in this study are listed in Table 1. To generate the pVB_*sctK* plasmid, the *sctK* gene was amplified from EPEC genomic DNA using the primer pair sctK-NcoIF and sctK-H3R (Table 3). The resulting PCR fragment was cloned into the NcoI and HindIII sites of plasmid pBADMycHisA. Similarly, the pNB_*sctI* plasmid was constructed by amplifying the *sctI* gene using the primers sctINcoIFw and sctIHindIIIRv. The resulting PCR fragment was cloned into the NcoI and HindIII sites of plasmid pBADMycHisA. The pST_*sctI* plasmid was generated by subcloning *sctI* from pJE_*sctI* (26) into the NdeI and BamHI sites of pTrc99A_FF4. The pFT_*map* plasmid was generated by amplifying the *map* gene from EPEC genomic DNA using the primers MapF and MapR. The resulting PCR fragment was first cloned into the NdeI and HindIII sites of pET19b. Then, pFT_*map* was generated by subcloning the *map* gene from pNE_*map* into the NdeI and BamHI sites of pTrc99A_FF4. The pBT_*sctF* and pNE_*sctF* plasmids were generated by amplifying the *sctF* gene from EPEC genomic DNA using the primers SctF-F and SctF-R. The PCR fragment was then cloned as an NdeI and BamHI fragment into plasmid pTrc99A_FF4 or pET19b, respectively. The pJH_*sctF* plasmid was generated by amplifying the *sctF* gene from EPEC genomic DNA using the primers SctFforHindIII and SctFRevXhoI. The resulting PCR fragment was cloned into the HindIII and XhoI sites of pTOPO-2HA. To generate the pAT_*sctL* plasmid, the *sctL* gene was PCR-amplified from EPEC chromosomal DNA using the primers orf5BamF and orf5SalR. The PCR product was first cloned into the PCR-Blunt-II TOPO vector according to the manufacturer’s instructions (Invitrogen). Then, pAQorf5 was generated by subcloning *sctL* from pARorf5BS into the BamHI and PstI sites of pQE30. pAT_*sctL* was generated by subcloning *sctL* from pAQorf5 into the BamHI and PstI sites of pTrc99A. The pIT_*sctQ* plasmid was generated using Gibson assembly (84). The *sctQ* gene was amplified with primers Gib_sctQ_pTrc_f/ Gib_sctQ_pTrc_r from EPEC genomic DNA. The pTrc99AFF4 backbone was PCR-amplified with the primers Gib_uni_pTrc_f/ Gib_uni_pTrc_r using the pTrc99AFF4 plasmid as a template. Then, the PCR products *sctQ* and pTrc99AFF4 were assembled using the Gibson Assembly Cloning kit (New England Biolabs) following the manufacturer’s instructions. All constructs were verified by DNA sequencing at the Unidad de Biología Molecular, Instituto de Fisiología Celular, UNAM.

### Secretion assay

Overnight LB cultures of EPEC were diluted 1:50 in 6 mL of pre-warmed DMEM supplemented with 1:100 of LB. Cultures were grown at 37°C with 5% CO_2_ to an OD_600_ of ~0.8–1. Bacterial cells were harvested by centrifugation at 21,130 × *g* for 2 minutes at 4°C, and the bacterial pellet was stored at −20°C. The supernatant was subjected to a second round of centrifugation. Proteins in the supernatant were precipitated overnight with trichloroacetic acid (TCA; 10% [vol/vol]) at 4°C. Precipitated proteins were recovered by centrifugation at 21,130 × *g* for 30 minutes at 4°C. Both the bacterial and the precipitated supernatant protein pellets were resuspended in 1 x SDS sample buffer, with protein concentration normalized according to the OD_600_.

### Co-immunoprecipitation assay

Overnight LB cultures of EPEC were diluted 1:50 in 50 mL of pre-warmed DMEM supplemented with 1:100 of LB and 0.1% [wt/vol] arabinose. Cultures were grown to an OD_600_ of ~0.8. Bacterial cells were harvested by centrifugation at 4,516 × *g* for 15 minutes at 4°C, and the supernatant was discarded. The bacterial pellet was washed with 20 mL of ice-cold phosphate-buffered saline (PBS). The pellet was resuspended in 10 mL of buffer TNG (50 mM Tris, 150 mM NaCl, 1 mM EDTA, 20% [vol/vol] glycerol, and pH 7.5), and the bacterial cells were disrupted by sonication. The lysates were centrifuged at 12,857 × *g* for 15 minutes at 4°C, and the resulting supernatant was ultracentrifuged at 90,619 × *g* for 1 hour at 4°C. The clarified supernatant was incubated overnight with 40 µL of ANTI-FLAG M2 affinity gel (Sigma-Aldrich) with constant shaking at 4°C. Samples were centrifuged at 5,000 × g for 30 seconds, allowing the resin to settle for 2 minutes, and washed eight times with 1 mL of washing buffer (50 mM Tris, 150 mM NaCl, pH 7.5). Proteins were eluted from the resin with 0.5 column volumes of 2 x SDS sample buffer.

### Immunoblotting

Protein samples were loaded onto 15% SDS-PAGE gels and subsequently transferred onto PVDF or nitrocellulose membranes. The membranes were blocked overnight with 5% wt/vol of non-fat milk in TTBS buffer (Tris 20 mM, NaCl 150 mM, pH 7.4, Tween-20 0.1% vol/vol). Immunodetection was carried out with commercial antibodies α-HA (Roche), α-DnaK (Enzo), α-c-Myc (Sigma-Aldrich), and α-FLAG (Roche) as well as polyclonal antibodies against the protein of interest. When needed, a secondary antibody HRP-conjugated anti-rabbit (Santa Cruz) or anti-mouse (Santa Cruz) was used. Detection of protein bands was performed using the Western Chemiluminescent HRP Substrate kit (Millipore). For immunoblotting against the SctF protein, the C-DiGit Chemiluminescent Western Blot Scanner from LI-COR was used.

## Data Availability

The data presented in this study are available in this article and its supplementary materials.

## SUPPLEMENTAL MATERIAL

Supplemental figures jb.00446-24-s0001.pdf Figures S1 to S6.

ASM does not own the copyrights to Supplemental Material that may be linked to, or accessed through, an article. The authors have granted ASM a non-exclusive, world-wide license to publish the Supplemental Material files. Please contact the corresponding author directly for reuse.
