# Supplementary material for: On the role of the sorting platform in hierarchical type III secretion regulation in enteropathogenic Escherichia coli
Source: J Bacteriol. 2025 Mar 3;207(3):e00446-24. doi: 10.1128/jb.00446-24 (PMC11925242; doi:10.1128/jb.00446-24)
Supplement: Supplemental figures — Figures S1 to S6. [file jb.00446-24-s0001.pdf]

On the role of the sorting platform in hierarchical type III secretion regulation in enteropathogenic *Escherichia coli*

Arely Marcos-Vilchis, Norma Espinosa, Adrián F. Alvarez, José L. Puente, J. Eduardo Soto, Bertha González-Pedrajo

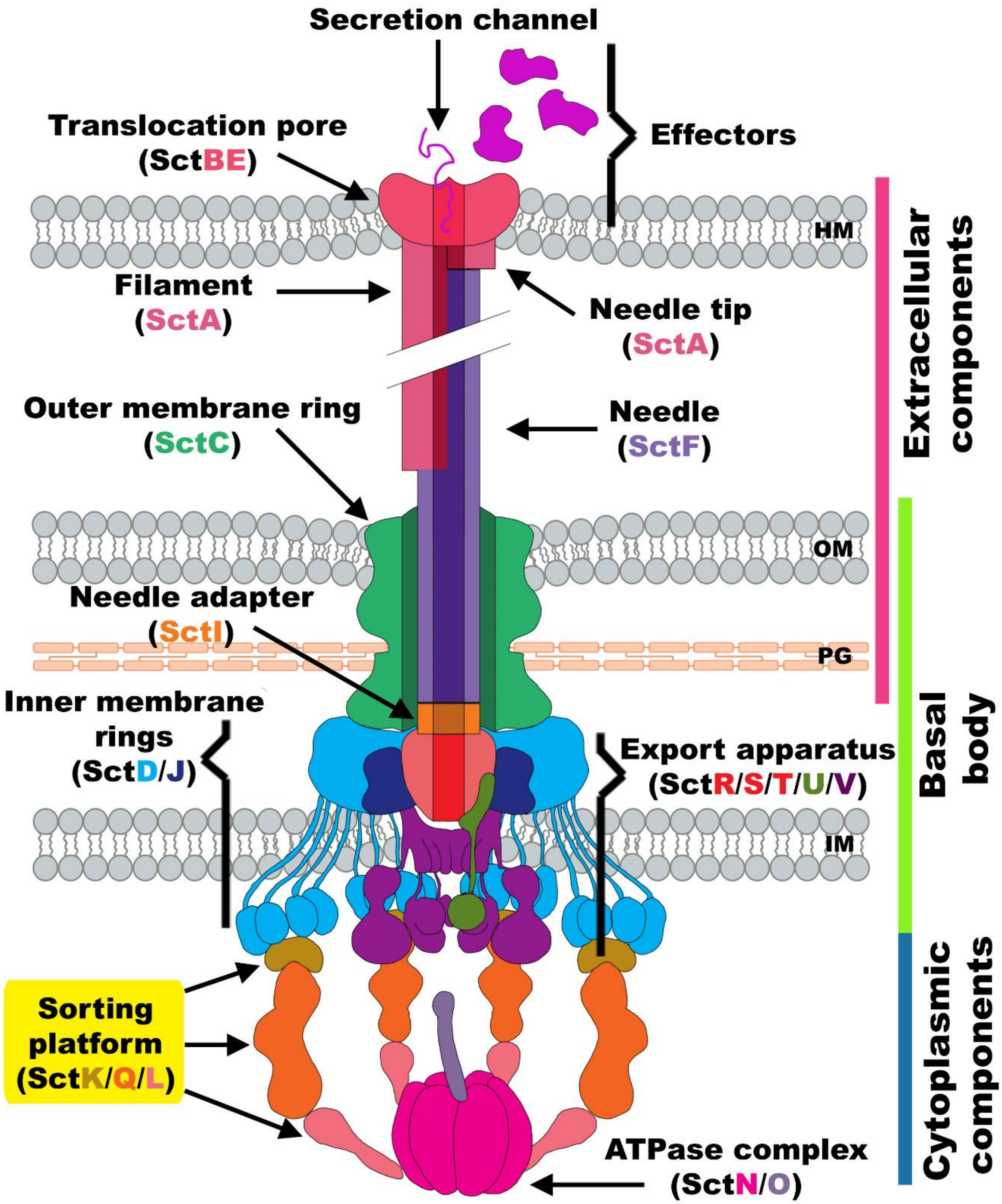

Supplementary Figure 1. Schematic diagram of the T3SS structure. Each structural component of the T3SS is labeled using the *sct* unified nomenclature. An unfolded late substrate being transported through the secretion channel is also depicted. The filament of A/E pathogens is schematized on the left side and the needle tip of many other bacterial pathogens on the right side. The relevant proteins studied in this work (SctK, SctQ, SctL) are highlighted within a yellow rectangle. HM: host membrane; OM: outer membrane, PG: peptidoglycan; IM: inner membrane.

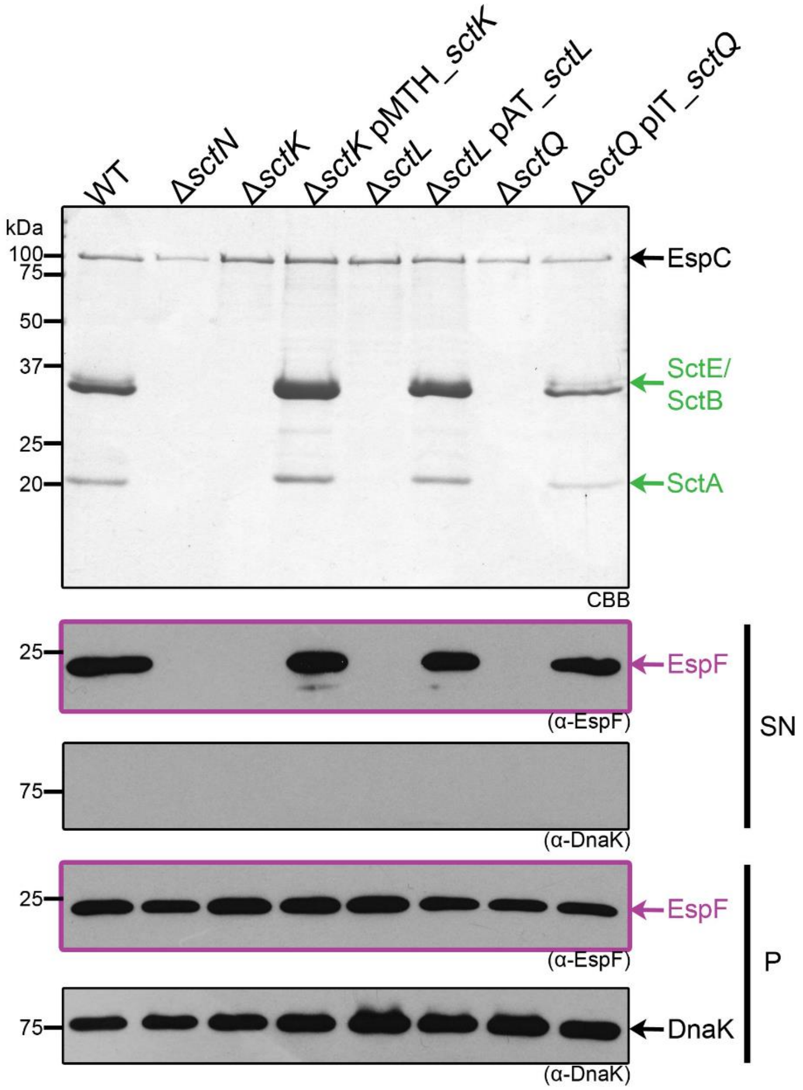

Supplementary Figure 2. Secretion phenotype of the non-polar SP mutant strains used in this study. Wild-type EPEC (WT) or the indicated non-polar mutant strains carrying a plasmid expressing His-SctK (pMTH_*sctK*), untagged SctL (pAT_*sctL*), or untagged SctQ (pIT_*sctQ*), were grown under T3S-inducing conditions. Secreted proteins in the culture supernatant (SN) or whole-cell lysate (P) were detected by either Coomassie brilliant blue (CBB) staining or immunoblotting against EspF or DnaK. Anti-DnaK antibodies served as a loading control for bacterial whole-cell lysates and as a cell lysis control in the bacterial supernatant. Substrates are color-coded: middle substrates in green and late substrates in magenta. Results are representative of three independent biological replicates. Molecular mass markers are shown on the left (kDa).

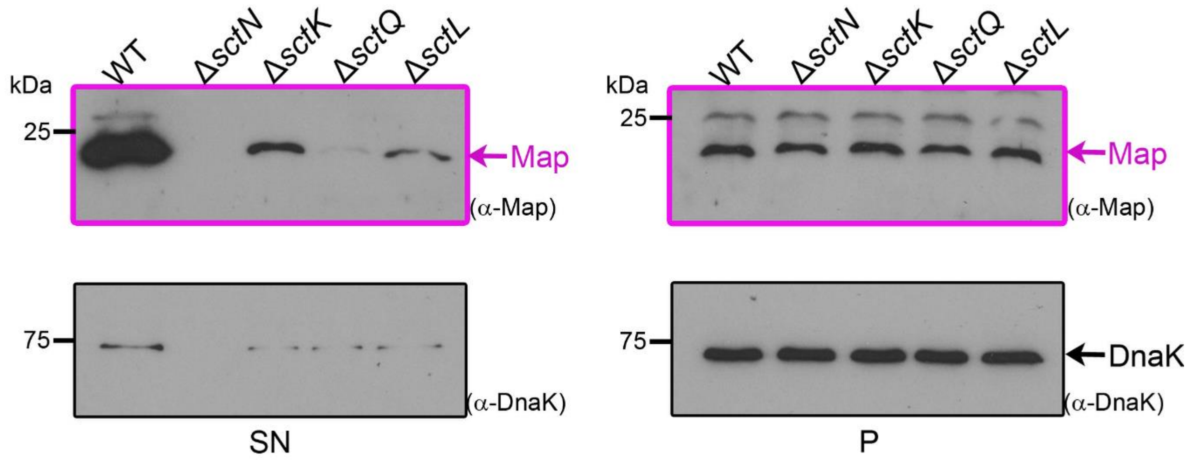

Supplementary Figure 3. Bypass of Δ*sctK* and Δ*sctL* strains by late substrates overproduced from the pTrc99A_FF4 vector. The indicated EPEC strains carrying a pTrc99A_FF4-based construct expressing the untagged effector *map* (pMT_*map*) were grown in T3S inducing conditions. Secreted proteins were detected in the culture supernatant (SN) or whole-cell lysate (P) by immunoblotting against the late substrate Map (magenta) or DnaK. Anti-DnaK antibodies served as a loading control for bacterial whole-cell lysates and as a cell lysis control in the bacterial supernatant. Molecular mass markers are shown on the left (kDa).

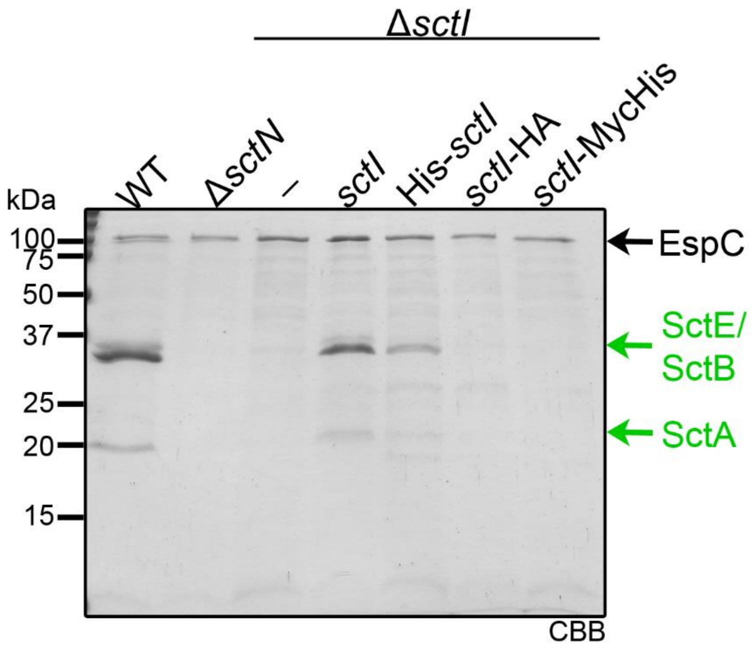

Supplementary Figure 4. Epitope tags impair SctI function. Wild-type EPEC (WT) and the indicated non-polar *sctN* or *sctI* null mutant strains expressing untagged *sctI* (pST_*sctI*), His-*sctI* (pJE_*sctI*), *sctI*-HA (pJH_*sctI*), or *sctI*-MycHis (pNB_*sctI*) were grown under T3S-inducing conditions. Secreted proteins were recovered, separated by SDS-PAGE and visualized using Coomassie brilliant blue (CBB) staining. The bands corresponding to the middle substrates SctB, SctE, and SctA are highlighted in green. Molecular mass markers are shown on the left (kDa).

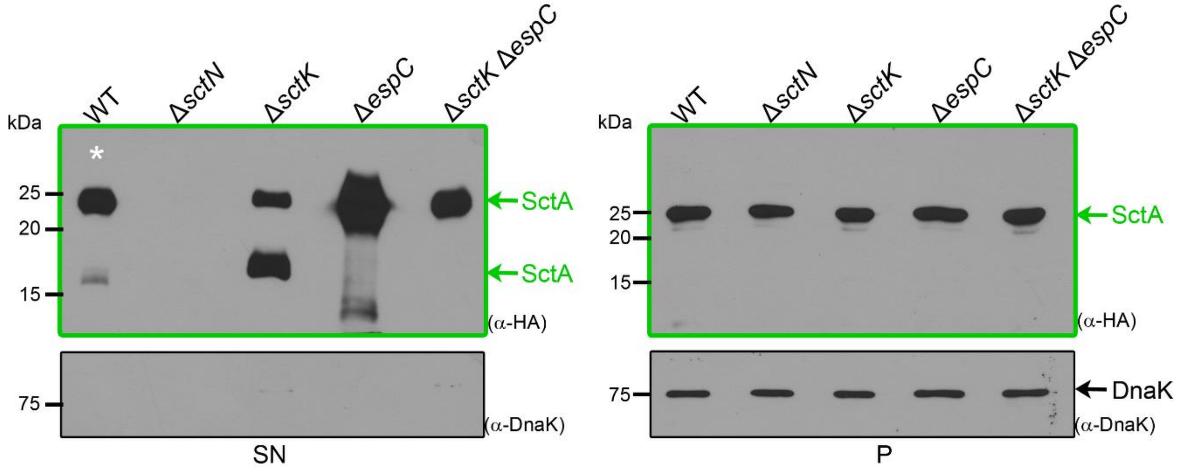

Supplementary Figure 5. The middle substrate SctA is specifically processed by the serine protease autotransporter EspC. Wild-type EPEC (WT) or the indicated non-polar mutant strains carrying a plasmid expressing SctA with a C-terminal double hemagglutinin (2HA) epitope tag (SctA-HA) were grown under T3S-inducing conditions. Secreted proteins were analyzed in the culture supernatant (SN) or whole-cell lysate (P) by immunoblotting against the HA tag or DnaK. To prevent signal saturation, the amount of supernatant sample loaded for the WT strain lane was diluted 1:12 (*****). The bands corresponding to the middle substrate SctA are highlighted in green. EspC preferentially promotes proteolysis of unassembled SctA, which is more abundant in the Δ*sctK* mutant than in the WT strain (compare lanes WT and Δ*sctK*). Molecular mass markers are shown on the left (kDa). The results are representative of three independent experiments.

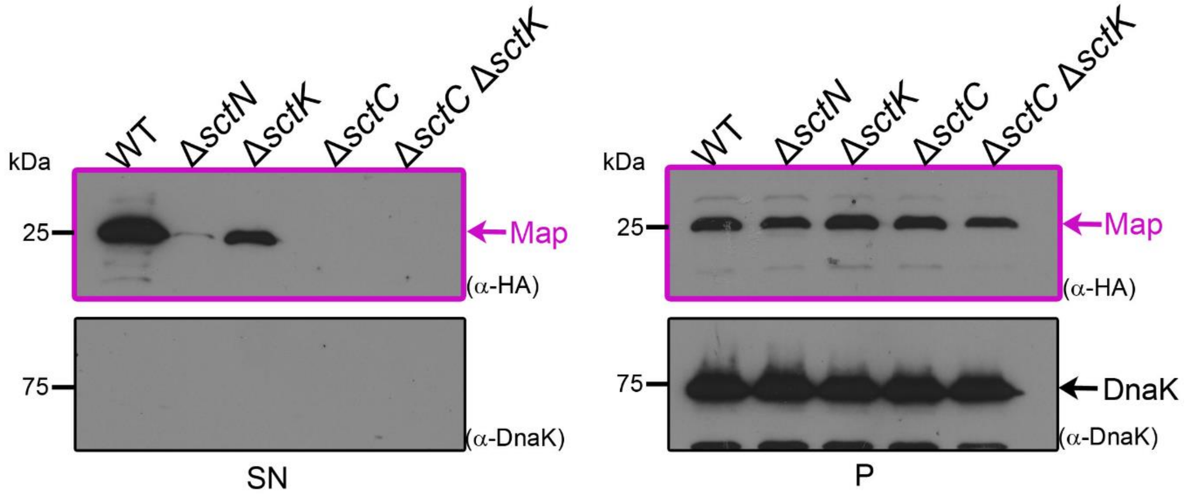

Supplementary Figure 6. Bypass of the Δ*sctK* **mutant by overproduction of the late substrate Map requires the secretin SctC.** The indicated EPEC strains carrying a plasmid expressing Map with a C-terminal double hemagglutinin (2HA) epitope tag (Map-HA) were grown under T3S-inducing conditions. Secreted proteins were analyzed in the culture supernatant (SN) or whole-cell lysate (P) by immunoblotting against the HA tag or DnaK. Molecular mass markers are shown on the left (kDa). The results are representative of two independent experiments.
